# Fowler syndrome post liposculpture and augmentation mammoplasty: Case report and literature review

**DOI:** 10.1016/j.jpra.2024.09.013

**Published:** 2024-09-26

**Authors:** Rodrigo Domínguez-Millán, Tatiana Luna Pisciotti, Pamela Fernández Cuesta, Miguel Angel Bonilla Becerril

**Affiliations:** a Private Practice, Corporea Health & Aesthetics, Mexico City, Mexico; b Pontificia Universidad Javeriana, Bogotá D.C., Colombia; c Universidad Panamericana, Mexico City, Mexico; d Private Practice, American British Cowdray Medical Center, Mexico City, Mexico

**Keywords:** Fowler's syndrome, Liposuction, Mammoplasty

## Abstract

Fowler syndrome is a rare entity that occurs in young women, with a high incidence of polycystic ovary syndrome. It consists of episodes of acute urinary retention that have a characteristic electromyographic pattern. Its etiology is unclear and is usually preceded by a trigger such as a surgical procedure, childbirth, other acute medical conditions, and opioid use. There is no report in the literature that links any Plastic Surgery procedure to Fowler Syndrome. We present the case of a 31-year-old female patient who had an episode of acute urinary retention 36 hours after liposculpture and augmentation mammoplasty. Infectious, anatomical and functional causes were ruled out upon admission to the emergency room, confirming the diagnosis of Fowler Syndrome. The patient was treated with bladder catheterization for 72h and tamsulosin for 15 days, with adequate response. Subsequent to this she presented a favorable evolution without new episodes of difficulty for urination, without functional sequelae or complications in the aesthetic surgical result.

## Introduction

Fowler syndrome is a rare condition that causes acute urinary retention. It occurs in young women with a high incidence of polycystic ovary syndrome and is associated with triggering factors such as some surgical procedures (mainly gynecological), childbirth or other acute conditions.^1^ We present a case of Fowler Syndrome in a young female patient after an aesthetic surgical procedure.

## Case presentation

A 31-year-old female patient with history of gastritis and recurrent colitis with medical management, as well as cervical conization 9 years ago, without other important gynecological or surgical history, was programmed for high definition liposculpture and augmentation mammoplasty.

The procedure was performed under general anesthesia, with placement of a Foley catheter at the beginning of the procedure. Moderate definition liposculpture^2^ was performed on arms, anteroposterior trunk and thighs with VASER and Microaire technology. A total of 3,700 cc of Hartmann solution + epinephrine 1mg/1ml was infiltrated and a total of 2,600 cc was aspirated. Subsequently, gluteal fat transfer and hip expansion were performed injecting 900 cc of fat bilaterally, subcutaneously. Finally, a subglandular augmentation mammoplasty was performed with Motiva Ergonomix 230 cc implants. The surgery lasted 4 hours and was completed without complications.

According to the senior author´s liposculpture protocol, the patient remained hospitalized one night with an analgesic infusion pump with tramadol, metamizole, ondansetron, and dexmedetomidine. The patient referred a mild burning pain in the pubic region as the only discomfort during the postoperative hours. The Foley catheter was removed 24 hours post procedure, clear spontaneous micturition was confirmed before patient discharge.

Approximately 12 hours after discharge, the patient was admitted to the emergency department due to a disabling pubic pain, decreased urinary output and bladder tension, which progressed to urinary retention. She was evaluated by the urology department who performed urinary catheterization without complications, draining 1200 cc of clear urine. Urinary analysis and bladder structural ultrasound were performed which ruled out urinary tract infection and a possible bladder perforation caused during liposculpture. Given the context of a young patient with an episode of acute urinary retention after a surgical procedure and having other etiologies ruled out, a diagnosis of Fowler Syndrome was made.

The bladder catheter was removed 72 hours after, spontaneous micturition was present and the patient was discharged with outpatient surveillance and treatment with tamsulosin for 15 days. Follow-up appointments showed favorable patient evolution without new episodes of urination retention, functional sequelae, or compromise of the aesthetic surgical result.

## Discussion

Acute urinary retention is a rare entity whose incidence increases in the surgical population (2.0%-3.8%).^3^ It may be secondary to anatomical, neurogenic, myogenic factors, use of drugs such as anticholinergics, opioids and antidepressants, and in some cases may be idiopathic.^1^

Among the causes of acute urinary retention is Fowler Syndrome, first described in 1986.^4^ Clinically it manifests as an episode of non-painful acute urinary retention with a high residual volume of urine (>1000mL), severe pain when removing the bladder catheter and a characteristic electromyographic pattern, consisting in repetitive discharges and decelerating bursts.^5^ It occurs in young women in the second and third decades of life and has a variable association with polycystic ovary syndrome.^6^ Most have a history of abnormal pre-event urination habits, given for extended periods between urination and intermittent urination. The episode of acute urinary retention is usually preceded by a triggering factor, such as a surgical procedure, most often gynecological (30%),^7^ postpartum and other acute medical conditions.^1^

The pathological mechanism of Fowler syndrome consists of an increase in the afferent activity of the urethra with consequent inhibition of the afferent signals of the bladder towards the central nervous system. This results in an abnormal response of the brain to bladder filling, with impossibility for sphincter and urethral relaxation, inhibiting the onset of urination^5^^,^^7^ The symptoms may be exacerbated by the use of opioids, which have a similar mechanism by raising urethral pressure, inhibiting bladder reflex.^1^

Fowler Syndrome is an exclusion diagnosis. It should be suspected in any young woman who has an episode of acute urinary retention, having ruled out other more common etiologies with a full evaluation of the urinary tract and its neural function. Electromyography is the study of choice for the diagnosis of this entity, however it is painful, presents a high rate of false positives, is operator dependent and requires specialized equipment for its realization.^1^

The management of Fowler syndrome is mainly based on intermittent bladder catheterizations. These can be painful due to the contraction of the detrusor at the passage of the catheter. No medications have been reported to improve bladder function in Fowler syndrome, nor have results been seen with the use of intra-sphincter botulinum toxin.^1^ The only intervention that has shown to restore bladder function in women with Fowler syndrome is sacral neuromodulation by reinstating afferent signals to the brain, effective up to 75%.^5^^,^^8^

## Conclusions

As far as we have been able to find, prior to this publication, there are no previously reported associations between Fowler Syndrome and plastic surgery procedures in literature. We consider the information in this case useful as a clinical episode in which the first diagnosis to rule out would be serious complications caused by liposuction such as direct bladder injury by perforation. We suggest that in cases of urinary retention after liposuction or breast procedures in young patients where there is no evidence of direct bladder trauma or a urinary tract infection, Fowler Syndrome should be taken into account as a possibility, which will be easily managed, without functional or aesthetic consequences for patients.

## Informed consent

The patient consented to the publication of the case.

## Ethical approval

Not required.

This manuscript was adhere to the STROBE guidelines.

## CRediT authorship contribution statement

**Rodrigo Domínguez-Millán:** Conceptualization, Investigation, Project administration, Supervision, Writing – original draft. **Tatiana Luna Pisciotti:** Investigation, Writing – original draft. **Pamela Fernández Cuesta:** Investigation, Writing – review & editing. **Miguel Angel Bonilla Becerril:** Supervision.

## Declaration of competing interest

The authors report there are no competing interests to declare.
